# Current Status of Pancreas and Islet Cell Transplantation

**Published:** 2012-05-01

**Authors:** R. F. Saidi

**Affiliations:** *Assistant Professor of Surgery, Division of Organ Transplantation, Department of Surgery, University of Massachusetts Medical School, USA*

**Keywords:** Pancreas transplantation, Simultaneous pancreas-kidney transplantation, Diabetes mellitus, Glycemic control, Islet cell transplantation

## Abstract

Pancreas transplantation has emerged as an effective treatment for patients with diabetes mellitus, especially those with established end-stage renal disease. Surgical and immunosuppressive advances have significantly improved allograft survival. The procedure reduces mortality compared with diabetic kidney transplant recipients and wait listed patients. Improvements in diabetic nephropathy and retinopathy have also been demonstrated. Pancreas transplantation can improve cardiovascular risk profiles, improve cardiac function and decrease cardiovascular events. Lastly, improvements in diabetic neuropathy and quality of life can result from pancreas transplantation. Pancreas transplantation remains the most effective method to establish durable euglycemia for patients with diabetes mellitus.

## INTRODUCTION

Diabetes mellitus afflicts about 6% of the general population; it is currently the third most common disease and the fourth leading cause of death by disease in the United States. Of the estimated 21 million diabetic individuals in the United States, 1 to 2 million has insulin-dependent diabetes mellitus (IDDM). Nearly 300,000 new cases of IDDM are diagnosed each year, and the incidence is increasing [1]. Pancreas transplantation has assumed an increasingly important role in the treatment of IDDM. It is currently the only available treatment that reliably provides an insulin-independent state, resulting in euglycemia and normal glycosylated hemoglobin levels by re-establishing endogenous insulin secretion responsive to normal feedback controls [1].

Pancreas transplantation was first described in 1967 [2], but the initial pancreas graft and patient survival rates were dismal. A variety of factors, including advances in surgical techniques, immunosuppression, graft preservation techniques, methods of diagnosis and treatment of rejection, and management of common post-transplantation complications, have led to significant improvements in graft and patient survival. As a result, the total number of pancreas transplant procedures reported to the United Network of Organ Sharing (UNOS) and the International Pancreas Transplant Registry (IPTR) continued to increase—a total of 26,571 from December 1966 to October 2002, with most (n=20,014) performed in the United States [3].

**Indications for and Types of Pancreas Transplantation**

Patients with type 1 diabetes and end-stage renal disease (ESRD) have the choice of three transplant procedures—kidney transplant alone, simultaneous pancreas-kidney (SPK) transplant, or kidney transplant followed by pancreas transplant (pancreas-after-kidney [PAK] transplant), where the kidney graft is obtained from either a living or deceased donor. The usual indication for this procedure at most centers is a type 1 diabetes patient with ESRD.

Simultaneous Pancreas-Kidney (SPK) Transplantation 

In SPK transplantation, the pancreas and kidney are usually obtained from the same deceased donor. Therefore, changes in kidney function can be used to determine whether rejection is occurring in either organ [4-6].

Pancreas-after-Kidney (PAK) Transplantation 

This is the second most common pancreas transplant procedure. The indication for this procedure is a patient with type 1 diabetes who has identified a living donor for kidney transplant and wants to plan a later PAK transplant, or the type 1 diabetes patient who already has a kidney transplant that has stable graft function, desires the potential benefits of euglycemia, and has the cardiac reserve to undergo the procedure [4-7].

Pancreas Transplant Alone (PTA) 

Pancreas transplant alone is the least common pancreas transplant procedure performed. Frequent, severe, hypoglycemic events are the most common indication for this procedure. The American Diabetes Association position statement suggests that indications for pancreas transplant (in the absence of kidney failure) are “frequent, acute and severe metabolic complications (hypoglycemia, hyperglycemia, and ketoacidosis) requiring medical attention” as well as “clinical and emotional problems with exogenous insulin therapy that are so severe as to be incapacitating; and consistent failure of insulin-based management to prevent acute complications” [4].

**Figure 1:**
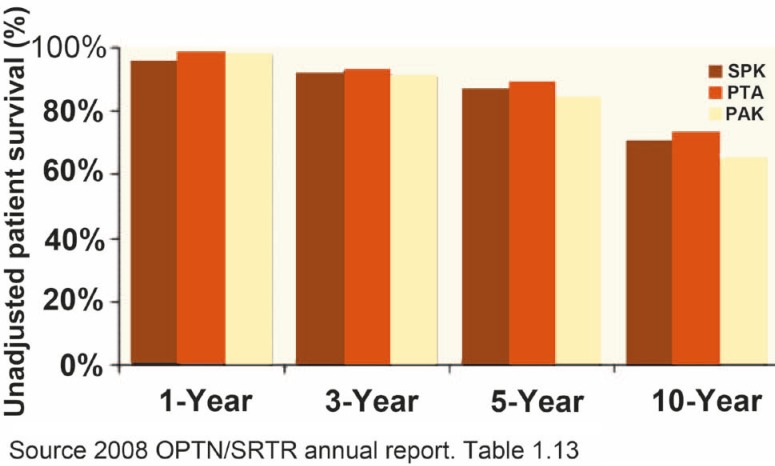
Unadjusted 1-, 3-, 5- and 10-year patient survival after pancreas transplantation stratified by transplantation type

**SURGICAL ISSUES IN PANCREAS TRANSPLANTATION**

Bladder vs. Enteric Duct Management 

With bladder drainage (BD), urine amylase can be used as a marker of graft function. Biopsies of the pancreas graft are also easily obtained across the bladder wall through cystoscopy. However, this procedure also creates potential complications. Metabolic acidosis occurs in most cases, and extracellular volume depletion is common, occasionally severe enough to require hospitalization; both complications are due to the loss of sodium bicarbonate-rich pancreatic secretions into the urine. Additional problems that can complicate BD include bladder leak, reflux pancreatitis, particularly with neurogenic bladder, chemical cystitis/urethritis, and frequent bladder infections, duodenitis in the connecting segment, bladder tumors, bladder calculi, urethral stricture, urethral erosion, epididymitis, prostatitis, and prostatic abscess. The frequency of urological complications is high (50%–77%), but they rarely result in graft or patient loss.

The alternative to BD is enteric drainage (ED) of the exocrine duct. In this procedure, the pancreatic duct is inserted into the small bowel using a small “button” of duodenum or with a Roux-en-Y limb. There is less need for monitoring the pancreas graft, overall, because immunosuppression has improved and frequency of rejection episodes has decreased after pancreas transplant of all types. Indications for enteric conversion surgery (20%–25%) are frequent episodes of severe extracellular volume depletion, severe metabolic acidosis, urological complications, or problems with the duodenal segment.

**Figure 2:**
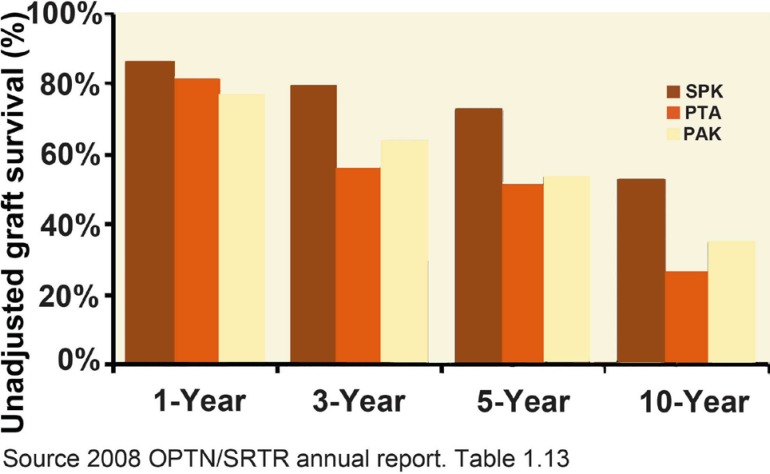
Unadjusted 1-, 3-, 5- and 10-year graft survival (death is included as an event) after pancreas transplantation stratified by transplantation type

SPK transplants performed with either BD or ED have equal pancreas graft survival.

Portal vs. Systemic Venous Drainage 

When placed in the systemic circulation—called systemic venous drainage (SVD)—the insulin secreted into the pancreatic venous effluent is not extracted immediately by the liver, as it would be if it emptied into the portal circulation. Systemic concentrations of insulin, both fasting and postprandial, are elevated as a result. Subsequently, a procedure was developed where the graft was placed in the portal circulation and the pancreatic duct was drained into the small intestine. This combined portal venous drainage (PVD) with ED procedure resulted in much lower peripheral insulin concentrations than pancreas transplant recipients with SVD, comparable to nondiabetic kidney transplants receiving similar immunosuppression.

**COMPLICATIONS**

The complexity of the whole pancreas transplant procedure, along with the likelihood of pre-existing disease secondary to their diabetes, exposes the recipient to a variety of significant operative and post-operative risks. The extent of the post-operative problems likely limited the widespread acceptance of pancreas transplantation in the early era of its development. Serious surgical complications following the procedure include thrombosis of graft vessels, intra-abdominal hemorrhage, anastomotic leak (enteric or bladder), graft pancreatitis, pancreatic fistula formation and intra-abdominal sepsis, all of which may require re-laparotomy and the possibility of graft loss. In recent years, with improvements in donor and recipient selection criteria, surgical technique, immunosuppression protocols (reduced incidence of early and acute rejection), and prophylaxis regimes (anti-viral, anti-bacterial and antithrombosis), there has been a significant decrease in the overall incidence of serious complications and the rate of re-laparotomy.

**IMMUNOSUPPRESSION**

The most common regimen in all pancreas transplant categories in 2000 was tacrolimus/mycophenolate mofetil (MMF, nearly 80%) with cyclosporine/MMF a distant second (5%–20%) [6]. The combination of tacrolimus/MMF has largely replaced cyclosporine/MMF because of some evidence of lower rejection rates, and better blood pressure and lipids. Other combinations are tacrolimus-sirolimus cyclosporine-sirolimus, tacrolimus-sirolimus-MMF, MMF-sirolimus, and sirolimus only.

Many centers still use corticosteroids in their immunosuppression protocol, which may allow a reduced dose of calcineurin inhibitor, but others have tried to move to a “steroid-free” protocol with the assumption that this will decrease risk of weight gain, glucose intolerance, dyslipidemia, and bone loss.

The results of clinical pancreas transplantation continue to improve as immunosuppressive strategies become more targeted. Although corticosteroid withdrawal or avoidance is a clinical reality, particularly in SPK and PAK recipients, CNI minimization or elimination remains a work in progress. Induction immunosuppression is routinely used in whole pancreas transplantation, with >75% of recipients receiving either 1) a T-cell depleting polyclonal antibody (thymoglobulin, ATGAM) or monoclonal antibody (OKT3, Campath); 2) a non-depleting monoclonal anti-CD25 antibody (Zenapax, Simulect); or 3) both. 

The initial results with alemtuzumab induction appear quite promising, although it appears to be more effective when used as a conventional induction agent rather than a tolerance-inducing or maintenance immunosuppressant. In SPK recipients, both T-cell depleting and non-depleting antibody agents for induction have been used, whereas in solitary PTX, the use of T-cell depleting antibodies has been greater. Monotherapy can be achieved in a proportion of patients, but it remains unclear which regimen is most effective and safe. Future clinical studies will continue to focus on long-term outcomes, appropriate donor and recipient selection, immunologic monitoring, immunosuppressive reduction, and outcomes-based research.

**RESULTS AND OUTCOME**

Whole-organ SPK transplant with normal graft function consistently improves 7- to 10-year patient survival compared with deceased donor kidney transplant, SPK transplant with loss of pancreas graft function, or dialysis in type 1 diabetic patients waiting for a transplant [3].

Patient survival after SPK transplant is consistently better than that observed after cadaveric-donor kidney transplant, with the possible exception of recipients over age 50. Although this advantage may, in part, be due to improved glucose after SPK transplant compared with kidney transplant alone, differences between the recipients who undergo these procedures, and between the donor grafts used for these two procedures, would likely also contribute to the difference in survival described between these two procedures. Mortality after SPK transplant is equal to living-donor kidney transplant alone after 10 years, and both PAK and pancreas transplant alone may increase 4-year mortality compared with remaining on the waiting list for those procedures. In these cases, specific quality of life (QOL) concerns and impact of pancreas transplant on specific diabetic complications need to be weighed against potential early increase in mortality before these procedures are considered.

Whole pancreas transplantation has proven to be a safe procedure with a 1- and 3-year patient survival rates for all forms of pancreas transplant (SPK, PAK, PTA) in the US since 1998 at almost 95%, and 89%, respectively (unadjusted patient survival rates, 2009 OPTN/SRTR Annual Report) [3].

Serum glucose level normalizes immediately after pancreas transplant, at the expense of hyperinsulinemia if SVD is used. Insulin secretion demonstrates oscillations despite denervation, as well as normal first- and second-phase secretion responses, unless there is a decrease in graft function or increased insulin resistance. C peptide concentrations are often slightly elevated, both basally and after mixed meal stimulus, but similar to those of non-diabetic kidney transplant recipients. Although fasting proinsulin is increased, it does not necessarily represent failing graft function, and glucagon response to hypoglycemia improves over time. Glucagon and symptom response to hypoglycemia return to normal or near normal over time; epinephrine and growth hormone responses though improve do not become normal after pancreas transplant. Hypoglycemic symptoms and documented events are uncommon and tend to diminish over time but may be due to a variety of factors. It should be cautioned that glucagon secretion in response to hypoglycemia does not improve with either allo- or auto-transplantation of islets into the liver, in human or animal studies, as described with pancreas transplant, and may be related to their location in the liver [8].

Diabetic nephropathy can be prevented by a functioning pancreas graft, and pathological changes of diabetes can reverse over time after more than five years of normal pancreas function [9]. Diabetic retinopathy may worsen initially after pancreas transplantation with sudden improvement in glucose concentration; therefore, evaluation and treatment of pre-existing retinopathy is important when pancreas transplant surgery is being considered [9, 10]. After three or more years of pancreas graft function, less retinal surgery is required after SPK transplant compared with kidney transplant alone in patients who do not already have end-stage eye disease. Lifelong eye surveillance examinations are required in all pancreas transplant recipients as laser surgery may still be required, particularly early after transplant surgery. Also, screening ophthalmic examinations are needed to evaluate cataracts that can form or progress, particularly in any patient treated with corticosteroids. Improvements in sensory and motor neuropathy occur after both SPK transplantation and kidney transplant alone. However, greater improvements have been reported after SPK transplant with ongoing improvements up to 10 years after the transplant. Autonomic neuropathies may take longer to improve (10 years or more) and may be only partially reversible or not reversible at all in some cases. Yet some autonomic neuropathy parameters are improved in some studies, particularly hypoglycemia awareness, autonomic response to hypoglycemia, and cardiac autonomic neuropathies [11, 12].

The most common cause of death in diabetes and transplant patients is vascular disease. Improved glucose control, as with SPK transplant, can improve vascular reactivity and microvascular integrity and responses, but other factors after transplant may prevent or minimize these improvements in some patients [11]. Changes in renal function; genetic predisposition to hypertension, dyslipidemia, or insulin resistance; the types of immunosuppressants used and their relative dose; changes in behavior as with weight gain or smoking cessation; and even donor and graft variables that contribute to delayed or decreased renal function, or frequency of rejection that increases the need for immune suppression may all impact risk over time. However, the results to date suggest that macrovascular disease improves in most patients after SPK transplant, but inadequate data are available to comment on change in risk after pancreas transplant alone or PAK transplant.

**ISLET CELL TRANSPLANTATION**

Islet cell transplantation is an attractive alternative therapy to conventional insulin treatment or vascularized whole pancreas transplantation for type 1 diabetic patients by islet cells isolation from donor pancreata and embolization into the recipient liver via the portal vein. Compared to pancreas transplantation, islet cell transplantation is technically much simpler (although there are still potential risks), has lower morbidity and offers the opportunity for storage of the islet graft in tissue culture or cryopreservation for banking [12-15].

With recent advances in methods of islet isolation and the introduction of more potent and less diabetogenic immunosuppressive therapies, islet cell transplantation has progressed from bench to clinical reality. Presently, several international centres have demonstrated successful clinical outcomes with high rates of insulin independence after islet transplantation [15]. Ongoing refinements in donor pancreas procurement and processing, developments in islet isolation and purification technology, and advances in novel immunological conditioning and induction therapies have led to the acceptance of islet transplantation as a safe and effective therapy for patients with type 1 diabetes.

Over the past few years, there has been tremendous progress in clinical islet transplantation, from refinements of the Edmonton Protocol to novel strategies for improved islet isolation, implantation and recipient immunosuppression. One of the most critical areas of research is the islet isolation procedure, which remains highly labor intensive, expensive and relatively inconsistent. Even the highest grade preparations only recover about 20%–50% of the potential islet mass. Moreover, rates for successful islet isolation at leading centers vary from 25% to 75%, depending largely on the quality of the pancreas, the amount of cold storage and the heterogeneity of collagenase preparations [12-15].

As islet transplantation moves forward, one of the first challenges is to reliably achieve insulin independence with single-donor grafts. Based on experience with islet auto-transplantation after total pancreatectomy, a minimum of 300,000 islets are necessary to achieve insulin independence in 70% of recipients [15]. This is in stark contrast to the 850,000 islets required in the Edmonton series of patients, suggesting that factors such as the presence of autoimmunity, diabetogenic immunosuppression, and brain death of the donor may have detrimental effects on islet engraftment and function. Cadaveric brain-dead organ donors are often hemodynamically unstable, requiring high doses of inotropic support; circulating brain-derived inflammatory peptides can also have direct toxic effects on the pancreas prior to retrieval. Advances in procurement techniques from cadaveric donors and improvements with less toxic and more potent immunosuppression will progressively lead to lower islet requirements to achieve euglycaemia.

Development of novel immunosuppressive protocols using more specific and less toxic drugs, ultimately towards inducing tolerance, is an important step in applying islet transplantation earlier in the course of the disease, including transplantation in children. Moreover, advances in identifying other sources of islet cells, together with progress in better understanding the biology of diabetes, will help increase the limited supply of islets through gene therapy, stem-cell biology techniques or xenotransplantation. It is anticipated that continued international collaboration will further stimulate excitement in the field, as innovative solutions are created to meet the remarkable challenges that lie ahead.

## References

[1] American Diabetes Association (2004). Pancreas transplantation for patients with type 1 diabetes. Diabetes Care.

[2] Kelly WD, Lillehei RC, Merkel FK (1967). Allotransplantation of the pancreas and duodenum along with the kidney in diabetic nephropathy. Surgery.

[3] McCullough KP, Keith DS, Meyer KH (2009). Kidney and pancreas transplantation in the United States, 1998-2007: access for patients with diabetes and end-stage renal disease. Am J Transplant.

[4] Rossi M, Lai Q, Spoletini G (2008). Simultaneous pancreas-kidney transplantation: a single-center experience and prospective analysis. Transplant Proc.

[5] Reddy KS, Stablein D, Taranto S (2003). Long-term survival following simultaneous kidney-pancreas transplantation versus kidney transplantation alone in patients with type 1 diabetes mellitus and renal failure. Am J Kidney Dis.

[6] Venstrom JM, McBride MA, Rother KI (2003). Survival after pancreas transplantation in patients with diabetes and preserved kidney function. JAMA.

[7] Humar A, Ramcharan T, Kandaswamy R (2004). Technical failures after pancreas transplants: why grafts fail and the risk factors–a multivariate analysis. Transplantation.

[8] Dean PG, Kudva YC, Stegall MD (2008). Long-term benefits of pancreas transplantation. Curr Opin Organ Transplant.

[9] Lipshutz GS, Wilkinson AH (2007). Pancreas-kidney and pancreas transplantation for the treatment of diabetes mellitus. Endocrinol Metab Clin North Am.

[10] Knight RJ, Zela S, Schoenberg L (2004). The effect of pancreas transplantation on peripheral vascular disease complications. Transplant Proc.

[11] Mai ML, Ahsan N, Gonwa T (2006). The long-term management of pancreas transplantation. Transplantation.

[12] Iwanaga Y, Sutherland DE, Harmon JV, Papas KK (2008). Pancreas preservation for pancreas and islet transplantation. Curr Opin Organ Transplant.

[13] Rivas-Carrillo JD, Okitsu T, Kobayashi N (2007). Current cell-based approaches for the treatment of diabetes mellitus. Biotechnol Genet Eng Rev.

[14] Hogan A, Pileggi A, Ricordi C (2008). Transplantation: current developments and future directions; the future of clinical islet transplantation as a cure for diabetes. Front Biosci.

[15] Ryan EA, Shandro T, Green K (2004). Assessment of the severity of hypoglycemia and glycemic lability in type 1 diabetic subjects undergoing islet transplantation. Diabetes.

